# Pyogenic Liver Abscess: Contemporary Trends in a Tertiary Institute

**DOI:** 10.1155/2022/4752880

**Published:** 2022-12-07

**Authors:** Elad Boaz, Eli Ben-Chetrit, Yonathan Bokhobza, Shlomo Yellinek, Menahem Ben-Haim, Petachia Reissman, Amir Dagan

**Affiliations:** ^1^Faculty of Medicine, Hebrew University of Jerusalem, Surgical Department, Shaare-Zedek Medical Center, Jerusalem, Israel; ^2^Faculty of Medicine, Hebrew University of Jerusalem, Infectious Diseases Unit, Shaare-Zedek Medical Center, Jerusalem, Israel

## Abstract

**Background:**

Pyogenic liver abscess (PLA) is an uncommon but potentially life-threatening condition. In recent years, advances in diagnostics and management have led to early diagnosis and treatment and decreased mortality. We present recent data from a large series of patients with PLA and examine the trends in the management of PLA over a period of 50 years.

**Methods:**

The medical records of all patients admitted to the Shaare Zedek Medical Center, Israel, between January 2011 and December 2021 with a primary or secondary diagnosis of PLA were reviewed retrospectively.

**Results:**

: Ninety-five patients with PLA were identified. Thirty-eight (40%) were female. The median patient age was 66 years (range 18–93). The diagnosis of PLA in all patients was confirmed with abdominal computed tomography (CT). In twenty patients (21.1%), PLA was not diagnosed by the initial abdominal US. Most abscesses were right-sided. Biliary tract origin was the most common underlying cause of PLA (*n* = 57, 60%), followed by cryptogenic etiology (*n* = 28, 30%). *Escherichia coli*, *Klebsiella pneumoniae,* and *Streptococcus* species were most commonly identified. The most common primary treatment modality was percutaneous drainage (PD), which was performed in 81 patients (85.3%). Fourteen patients (14.7%) were treated medically without intervention, and two patients (2.1%) were treated surgically following a failure of PD. Four patients died as a direct result of PLA.

**Conclusions:**

Patients diagnosed with PLA are older, the male predominance is less pronounced, and the offending pathogens are likely to originate from the biliary tract. This study questions the utility of abdominal US as the initial diagnostic imaging in patients with suspected PLA (versus CT) and demonstrates improved outcomes for patients with PLA over the years.

## 1. Introduction

Pyogenic liver abscess (PLA), while a relatively rare clinical condition with an incidence of 3.6/100,000 in the United States (US) [1], remains a clinical challenge owing to its occult clinical presentation and associated morbidity. In 1938, Ochsner et al. [2] described the management and outcomes of patients with PLA and recommended surgical treatment as the primary therapeutic modality. At that time, PLA was most commonly a complication of acute appendicitis and was associated with high mortality. Surgery remained the therapy of choice until the mid-1980s, when percutaneous drainage (PD) was shown to be a safer alternative [3–6]. With advances in diagnostic imaging and less invasive interventions, the mortality attributed to PLA has steadily declined. A recent nationwide study reported an inpatient mortality rate of 6% in the US [1], with international studies reporting patient fatality rates of 11–31% [7, 8]. Multiple studies have noted a gradual epidemiologic change with regard to age, clinical presentation, etiology, microbiology, and treatment strategies. These transitions have been attributed to better access to care, improved imaging techniques, increased frequency of biliary tract pathology, and early diagnosis and management of the classic disorders previously associated with PLA formation (e.g., appendicitis, diverticulitis) [8–10]. Indeed, the most common etiology of PLA reported in recent years has shifted from gastrointestinal sepsis to biliary disease [6–11]. Concerning microbiology, *Escherichia coli* and *Streptococci species* are the most common organisms associated with PLA in Western countries, whereas in Asia, *Klebsiella pneumoniae* and E. *coli* prevail [1, 12–14]. In this study, we present our current institutional experience in the management of PLA as compared to historical series [15, 16] in a similar patient population. Specifically, we aimed to describe the changes over time in the epidemiology, clinical presentation, management, and outcome of PLA over a period of 50 years.

## 2. Methods

The study was conducted at the Shaare Zedek Medical Center (SZMC), a tertiary teaching hospital in Jerusalem, Israel. All patients aged 18 or older who were diagnosed with PLA (based on discharge diagnosis) over a 10-year period from January 2011 to December 2021 were retrospectively identified. Medical files were retrieved and revised by the authors (EB, YB, and AD). Cases were included if the abscess was confirmed by imaging (either CT or US) and if there was documentation of an organism recovered from the abscess site or resolution of the abscess on imaging following antibiotic treatment. Patients with bacterial superinfection of an underlying tumor or cyst were excluded, as well as cases in which critical data were missing. Demographic, clinical, laboratory, microbiological, and radiologic data were collected and reviewed based on the patients' medical records. Those included ages, sex, ethnicity, year of admission, and underlying medical conditions (specifically biliary disease, appendicitis, diverticulitis, cholecystitis or other intraabdominal infections, malignancy, diabetes, hypertension, alcohol abuse, cardiovascular disease, and abdominal trauma). Presenting signs and symptoms were recorded, including fever, chills, right upper quadrant (RUQ) pain, nausea, vomiting, decreased appetite, weight loss, diarrhea, and jaundice, as well as laboratory parameters (complete blood count (CBC), lactate dehydrogenase (LDH) level, liver function tests, and C-reactive protein (CRP) level) on the day of admission. Imaging studies were reviewed to determine the location of the abscesses. Abscess size for each patient was determined as the largest diameter of the largest abscess as measured by ultrasound (US), computerized tomography (CT), or magnetic resonance imaging (MRI). Other imaging modalities that were performed, i.e., upper gastrointestinal series, colonoscopy, endoscopic retrograde cholangiopancreatography (ERCP), or percutaneous transhepatic cholangiography (PTC), were documented as well. The primary therapeutic approach was defined as PD or open surgery. If no intervention was performed, cases were classified as medically managed.

Defining the associated pathogen was based on positive blood cultures obtained during admission (BD, BACTEC™, USA) and/or abscess cultures (in the case of drainage). Antimicrobial susceptibility testing was performed using disk diffusion tests, or Etests (BD, USA) in accordance with CLSI guidelines [17]. When appropriate, serology for *Entamoeba histolytica* was sent to the Israeli Ministry of Health's reference laboratory for parasitic infections. The etiology of PLA was attributed to the process that was most likely to account for the abscess. If no clear cause was identified, the case was classified as cryptogenic. Other outcome measures that were addressed included antibiotic regimens, the duration of treatment, length of stay, in-hospital and 30-day mortality, and readmission secondary to PLA or its complications. A secondary intervention was defined as the need for drain manipulation, repeated aspiration, or surgery due to the progression or persistence of PLA (despite a primary intervention), as evidenced by clinical or radiographic features. Finally, the demographic and clinical characteristics of our cohort were compared to those of previous cohort studies in the same population [15, 16]. Statistical analysis was performed using SPSS software, version 24. Continuous variables were compared using analysis of variance, and categorical variables were compared with the chi-square test/Fisher's exact test. This study was approved by the institutional review board (approval number 0041-SZMC).

## 3. Results

**Figure 1:**
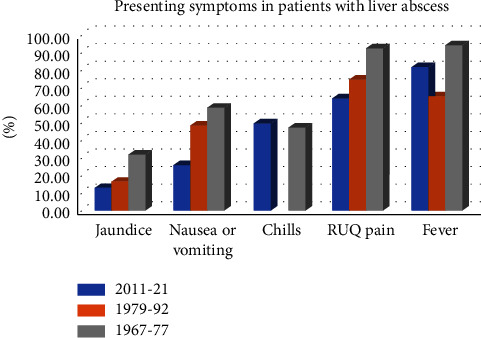
Presenting symptoms among patients with PLA in three consecutive cohorts in Jerusalem, Israel.

Nighty-five patients with PLA were identified between January 2011 and December 2021. The median age was 66 years (range 18–93). Thirty-eight (40%) were female. Seventy-eight patients (82.1%) were Jewish, 14 (14.7%) were Arabs, and 3 (3.2%) were from other ethnicities (Table 1). The median duration of symptoms was 3 days (IQR 5). The most frequent presenting symptoms and signs are shown in Figure 1. Fever (oral temperature above 38.1°C) and chills were reported in 77 (81.1%) and 47 (49.5%) patients, respectively. Among 60 (63.2%) patients, RUQ pain or tenderness was a prominent finding. The triad of fever, RUQ pain or tenderness, and an elevated alkaline phosphatase level was present in 26 (28.4%) patients. The most common laboratory abnormalities were increased WBC counts, elevated alkaline phosphatase, LDH, and CRP (Table 1). The diagnosis of PLA in all patients was confirmed with an abdominal CT. Of note, in twenty (21.1%) patients, PLA was not diagnosed by the initial abdominal US.  Table 2 shows selected clinical features of patients with a liver abscess in the current study as compared with those in the previous series from the same population [15, 16].

Most abscesses were located in the right lobe of the liver (*n* = 63, 66.3%). The most common underlying cause of PLA was biliary tract pathology (*n* = 57, 60%), followed by cryptogenic (*n* = 28, 29.5%), gastrointestinal (GI) pathologies (*n* = 8, 8.4%), and abdominal trauma (*n* = 2, 2.1%).

The most common primary treatment modality utilized was PD, which was performed in 81 patients (85.3%). Fourteen patients (14.7%) were treated medically without intervention, and two patients (2.1%) were treated surgically following a failure of PD. Patients treated medically had a median abscess size of 2.9 cm (range 1–9.2 cm), compared with 5.5 cm (range 1.5–23 cm) among the PD group. None of the patients who were treated medically required subsequent PD. The median duration of antibiotic therapy was 28 days (range 7–102 days). Of the 81 patients whose abscess was drained at some point during their treatment, 22 patients (27.2%) required drain replacement or repositioning due to diminished output, dislodgement, or clinical deterioration. Nine patients were readmitted due to abscess recurrence, of whom open surgical drainage was required in two. In both cases, the primary treatment modality was PD. The median length of stay (LOS) was 18 days (IQR 18.5) and was significantly longer for patients treated by PD compared with those treated only with medical therapy only (median of 20 days (range 6–31) vs. 10 days (range 5–75), respectively, *p*=0.007).

The overall mortality rate was 4.2% (*n* = 4). The median age of the patients who died was 85 (range 75–86). Three of them were female. The biliary disease was the presumed etiology for PLA in all four patients who died, and all were treated with PD.

**Figure 2:**
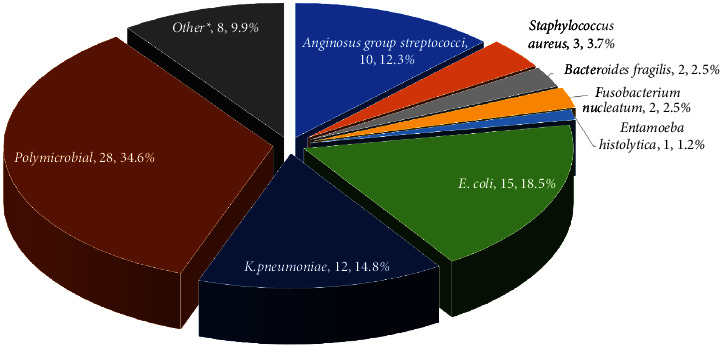
Pyogenic liver abscess organisms identified in our study ∗ other pathogens: *Pseudomonas aeruginosa*, *Klebsiella variicola*, *Haemophilus influenza*, *Enterococcus sp*., *Enterobacter sp*., *Citrobacter koseri*, *Candida albicans*.

Selected microbiological data are shown in Figure 2. Eighty-one patients had ≥1 organism recovered from the abscess. Twenty-eight (34.6%) of the infections were polymicrobial; in four cases (4.9%), anaerobic organisms were the only bacterial isolates. The most common pathogens were E. *coli,* K. *pneumoniae,* and S. *anginosus*. Other isolates included *Pseudomonas aeruginosa, Klebsiella variicola, Haemophilus influenza, Enterococcus sp., Enterobacter sp., Citrobacter koseri*, and *Candida albicans.*Fifty-two patients (55%) had concomitant bacteremia with the same pathogen isolated from the abscess. Among twenty-three isolates (all *Enterobacterales sp*.), multiple resistance patterns were noted (Table 3).

## 4. Discussion

The epidemiology, diagnosis, treatment, and mortality rate for patients with PLA have changed markedly over the past few decades. In this study, we present clinical data on PLA from three consecutive cohorts over a period of five decades, with several notable findings.

The age of patients diagnosed with PLA has increased substantially. In our study, the median age was 69 years (range 18–93), with 65.8% (65/95) of patients aged >60 years old and a slight male predominance. This finding was consistent with recent studies that also reported the presentation of symptoms at the ages of 55–65 years [1, 17–20]. These observations contrast with the early experience described by Ochsner et al. [2], where a younger age (20–50 years) and prominent male predominance (70%) were reported. These are likely related to changes in the etiology of PLA. The characteristic presenting symptoms were multiple and nonspecific, including fever, chills, and RUQ pain. With regard to laboratory findings, overall, the presence of elevated WBC, CRP, and alkaline phosphatase should suggest the possibility of PLA. In our study, as in other recent reports [12, 17–19], biliary disease was the most commonly identified cause of PLA. Portal spread, historically more common [2], has been replaced by biliary disease as the leading source of infection.

Abdominal US is often the initial radiological utility when PLA is suspected due to its good sensitivity (ranging from 67 to 97% [12, 21, 22]), availability, safety, low cost, and noninvasive nature. However, in our cohort, PLA was not diagnosed in 20 (21.1%) of the patients initially evaluated by the US, with a calculated sensitivity of only 62.3%. The relatively low sensitivity of abdominal US in our study cohort was concerning. The median size of the abscesses that were missed by the US was 4.5 cm (range 2–10), compared to 5.3 cm (range 2.9–23) in the rest of the cohort. CT has a 95% diagnostic accuracy for PLA [17, 23, 24] and was the most common imaging modality performed in this series (all patients). Given current CT availability and its sensitivity, our findings suggest that when PLA is suspected, CT imaging should be utilized earlier and PLA should not be ruled out by the US.

With regard to treatment, our findings are consistent with the trend toward a conservative and less invasive approach in the management of PLA. With a patient success rate of 85.3% with PD as the initial treatment, this study demonstrates that a PD-driven approach is both safe and effective. Antibiotics were given to all patients in this series, with fourteen patients (14.7%) treated exclusively with antibiotics with success. With a median size of 2.9 cm (range 1–9.2 cm), both single and multiple abscesses were successfully treated, as were those with singular and multiple organisms identified. Systemic antibiotic therapy (without drainage) has been previously shown to be an effective treatment for small PLA. Hope et al. [25] retrospectively reviewed 107 PLA cases and found that when PLA was smaller than 3 cm in diameter, antibiotic treatment alone was successful.

PD with concomitant antibiotics has been shown to be beneficial in the treatment of PLA and is the present standard of practice. Image-guided PD or aspiration was chosen over surgical drainage in the majority of cases in the current series, irrespective of the size of the abscess or the presence of multiloculations. However, other studies have shown varied results. In the study by Hope et al. [25], operative drainage was recommended in cases of large multiloculated abscesses. Tan et al., in their review comparing 36 patients undergoing PD to 44 patients who were treated surgically as first-line treatment, found that surgery was superior to PD in terms of success rate, number of secondary procedures, and hospital stay, with comparable morbidity and mortality rates to PD [26]. In contrast, Rismiller et al. [19] showed an 85% success rate with PD as the initial modality of treatment in 64 patients with PLA of varying size, and Liu et al. [27] similarly demonstrated successful PD in 109 patients with PLA, irrespective of size or loculation.

In our study, the yield from abscesses' cultures and blood cultures was 85.3% and 54.7%, respectively, which was higher than that described in other studies. This may be partially attributed to the early utilization of PD (and shorter exposure to antibiotics before cultures are attained). Regarding microbiology, 34.6% of the infections were polymicrobial. The predominant pathogens were E. *coli, Klebsiella sp*., and S. *anginosus* (Figure 2), which have also been reported in similar studies in western, Caucasian populations [1, 12, 13]. The large variety of bacterial isolates and the high percentage of polymicrobial infections support the use of empirical broad-spectrum antibiotics. This is a common practice in many institutions, pending culture results and antimicrobial susceptibility tests.

The in-hospital mortality rate in the present cohort was 4.2%. A much higher mortality rate (13%) was previously reported among patients from the same region [16], in which 19% underwent open surgical drainage. In our study, advanced age, septicemia with multidrug-resistant pathogens, and biliary etiology were risk factors for PLA-associated mortality.

Several limitations of this study should be noted. First, the data were gathered retrospectively; therefore, the accuracy of the data depends on the accuracy of record-keeping and, in some cases, fails to convey the nuanced factors contributing to a particular treatment plan. Furthermore, our results represent the experience of a single center; therefore, generalizations may be limited in terms of epidemiology and clinical setting.

In summary, we presented three consecutive cohorts of patients with PLA over a period of more than 50 years. Patients diagnosed nowadays with PLA are older, with a less prominent male predominance, and the likely cause of infection is biliary tract pathology. This study raises concern about whether abdominal US should be utilized as the initial diagnostic imaging tool in patients with suspected PLA. Finally, our findings support the conservative approach of PD and concomitant antibiotics (in contrast to operative drainage) as the mainstay of treatment of PLA[28, 29].

## Figures and Tables

**Table 1:** Demographic and clinical characteristics of patients with PLA in SZMC 2011–2021.

Characteristics	Median (range)
Age years (*n* = 95)	66 (18–93)
Female *n* (%)	38 (40%)
Duration of symptoms before admission, days (*n* = 90)	3 (1–31)
Length of stay according to treatment modality, days (*n* = 95)	
Percutaneous drainage and systemic antibiotics	20 (5–75)
Systemic antibiotics only	10 (6–31)
Duration of antibiotic therapy (overall), weeks	4 (1–12)
Duration of intravenous antibiotics, weeks	3 (1–12)
Duration of oral antibiotics, weeks	1 (0–4)
WBC count, mean ± SD (/mm^3^) (*n* = 95)	13.8 ± 6.3
Alkaline phosphatase, mean ± SD (*n*l 44–147 U/L) (*n* = 95)	234.6 ± 185.2
ALT, mean ± SD (*n*l 7–55 U/L) (*n* = 95)	55.8 ± 55.9
AST, mean ± SD (*n*l 8–48 U/L) (*n* = 95)	64.7 ± 84.1
LDH, mean ± SD (*n*l 105–333 IU/L) (*n* = 95)	397.2 ± 233.7
CRP, mean ± SD (*n*l 0.5< mg/dl) (*n* = 60)	20 ± 8.3

**Table 2:** Comparison of the clinical features, diagnosis, management, and outcome of PLA in Jerusalem.

Clinical parameters	2011–2021 (*n* = 95)	1979–1992 (*n* = 31)	1967–1977 (*n* = 36)
*Symptoms/signs*			
Fever> 37.6 C	77 (81.1%)	20 (65%)	34 (94%)
Vomiting	24 (25.3%)	9 (29%)	6 (6%)
Duration of symptoms (days)	5.5	6.4	96

*Etiology*			
Amoebic abscess	1 (1.2%)	1 (3%)	15 (42%)
Pyogenic abscess	79 (98.8%)	30 (97%)	21 (58%)
Anaerobic organisms	4 (4.9%)	5 (15%)	0

*Diagnosis*			
Noninvasive	95 (100%)	31 (100%)	21 (58%)
Surgical diagnosis	0	0	9 (25%)
Postmortem diagnosis	0	0	6 (17%)

*Management*			
Percutaneous drainage + antibiotics	81 (87.3%)	24 (77%)	2 (5%)
Percutaneous and open drainage	2 (2.1%)	5 (16%)	0
Open drainage only	0	1 (3%)	18 (50%)
Antimicrobial treatment only	14 (14.7%)	1 (3%)	10 (28%)
Survival	91 (95.8%)	27 (87%)	25 (69%)

**Table 3:** Resistance patterns among *Enterobacterales* isolates from liver abscesses.

Resistance patterns	*n* (%)
Wild type^*∗*^	42 (64.6)
ampC	7 (10.8)
CPE (kpc)	2 (3)
ESBL	14 (21.5)

^*∗*^ wild type refers to pan-susceptible E. *Coli* or K. *pneumonia* strains. CPE, carbapenemase-producing enterobacterales; ESBL, extended-spectrumbeta-lactamase.

## Data Availability

The data used to support the findings of this study are available from the corresponding author upon request.
